# Risk stratification of clinically relevant delayed gastric emptying after pancreaticoduodenectomy

**DOI:** 10.1186/s12893-023-02110-7

**Published:** 2023-08-09

**Authors:** Tian-Yu Li, Cheng Qin, Bang-Bo Zhao, Xiao-Ying Yang, Ze-Ru Li, Yuan-Yang Wang, Jun-Chao Guo, Xian-Lin Han, Meng-Hua Dai, Wei-Bin Wang

**Affiliations:** Department of General Surgery, State Key Laboratory of Complex Severe and Rare Diseases, Peking Union Medical College Hospital, Chinese Academy of Medical Sciences and Peking Union Medical College, Beijing, China

**Keywords:** Pancreaticoduodenectomy, Delayed gastric emptying, Risk factor, Risk model, Logistic regression

## Abstract

**Background:**

Delayed gastric emptying (DGE) remains one of the major complications after pancreaticoduodenectomy (PD), with discrepant reports of its contributing factors. This study aimed to develop a nomogram to identify potential predictors and predict the probability of DGE after PD.

**Methods:**

This retrospective study enrolled 422 consecutive patients who underwent PD from January 2019 to December 2021 at our institution. The LASSO algorithm and multivariate logistic regression were performed to identify independent risk and protective factors associated with clinically relevant delayed gastric emptying (CR-DGE). A nomogram was established based on the selected variables. Then, the calibration curve, ROC curve, decision curve analysis (DCA), and clinical impact curve (CIC) were applied to evaluate the predictive performance of our model. Finally, an independent cohort of 45 consecutive patients from January 2022 to March 2022 was enrolled to further validate the nomogram.

**Results:**

Among 422 patients, CR-DGE occurred in 94 patients (22.2%). A previous history of chronic gastropathy, intraoperative plasma transfusion ≥ 400 ml, end-to-side gastrointestinal anastomosis, intra-abdominal infection, incisional infection, and clinically relevant postoperative pancreatic fistula (CR-POPF) were identified as risk predictors. Minimally invasive pancreaticoduodenectomy (MIPD) was demonstrated to be a protective predictor of CR-DGE. The areas under the curve (AUCs) were 0.768 (95% CI, 0.706–0.830) in the development cohort, 0.766 (95% CI, 0.671–0.861) in the validation cohort, and 0.787 (95% CI, 0.633–0.940) in the independent cohort. Then, we built a simplified scale based on our nomogram for risk stratification.

**Conclusions:**

Our study identified seven predictors and constructed a validated nomogram that effectively predicted CR-DGE for patients who underwent PD.

## Introduction

Pancreaticoduodenectomy (PD) is one of the most challenging and complex surgeries in the abdominal surgery that carries a high rate of major complications, among which delayed gastric emptying (DGE) remains one of the most commonly observed complications with an incidence ranging from 15–35% [1–5]. Currently, the perioperative treatment of pancreaticoduodenectomy has entered the era of refined management, and the concept of enhanced recovery after surgery (ERAS) has been widely applied [6]. Although DGE is not a life-threatening complication, it can increase median hospital charges, prolong the length of hospitalization, increase the readmission rate and even affect long-term prognosis [7–11]. In recent years, many studies on the factors influencing DGE have been published. However, previous studies on its potential predictors were controversial and lacked reliable prediction models. Thus, it is essential to build a reliable predictive model for the prevention and treatment of DGE.

The definition of DGE has been inconsistently adopted in the previous literature. In 2007, a new grading system was proposed to define DGE and classify it as grades A, B, and C by the International Study Group of Pancreatic Surgery (ISGPS) [12]. In this article, the outcome variable was the incidence of grades B and C, also known as clinically relevant delayed gastric emptying (CR-DGE), which is defined as nasogastric tube required > 7 days, reinserted after POD 7, or unable to tolerate solid diet by POD 14 based on the ISGPS definition.

Nomogram is a graphical representation of complicated mathematical formulas that use clinical and biological variables to graphically portray a statistical predictive model, which can calculate the probabilities of clinical events for specific individuals [13]. The purpose of this study was to identify potential risk and protective factors of CR-DGE for patients undergoing PD, as well as establish and validate a scientific nomogram for doctors to conduct early intervention.

## Methods

### Patient selection

Our study retrospectively collected 422 consecutive patients undergoing pancreaticoduodenectomy from January 2019 to December 2021 in the Department of General Surgery of Peking Union Medical College Hospital, China. All patients had resectable pancreatic or periampullary tumor. Exclusion criteria were as follows: (1) patients with history of major abdominal surgery; (2) history of chronic organ insufficiency; (2) receiving surgery for non-neoplastic disease; (3) tumor invading the stomach; (4) intraoperative multiple-organ resection; (5) death during hospitalization or within 30 days after surgery; (6) delayed gastric emptying due to mechanical causes of abnormal gastric emptying, including postoperative mechanical intestinal obstruction, anastomotic edema, anastomotic stenosis. (7) confirmed distant metastases. (8) incomplete clinical data.

### Surgical procedure

In laparoscopic surgery, patients were positioned at 30 degrees reverse Trendelenburg’s position with split legs under general anesthesia. Then we elevated patients’ back by putting cushions under the xiphoid level. Five standard port sites were then adopted. In laparotomy or conversion to laparotomy, we chose a 20 cm right rectus abdominis incision. Lymph nodes were dissected in all patients, including No.8,12,13,14,17. After removal of the specimen, Child reconstruction was performed as follows. Firstly, an end-to-side duct-to-mucosa pancreaticojejunostomy, both the anterior and posterior wall of remnant pancreatic parenchyma was sewn to the seromuscular layer of the jejunum. For patients with a small pancreatic duct (≤ 3 mm), silicone stents were routinely inserted in the pancreatic duct and jejunum. Then, an end-to-side single-layer continuous choledochojejunostomy was performed 5-10 cm away from pancreaticojejunostomy. Lastly, the antecolic side-to-side or end-to-side gastrojejunostomy was constructed 30-40 cm away from the choledochojejunostomy. For end-to-side gastric-to-jejunal anastomosis, approximately 5–6 cm of the remnant gastric stump was anastomosed to the jejunal loop in a hand-sewn end-to-side fashion. For side-to-side anastomosis, the gastrojejunostomy was performed in the avascular area of posterior wall of the remaining stomach with the help of a linear stapler (Endo GIA 60 mm or Ethicon EC 60 mm), approximately 2 cm from the distal staple line. The direction of the anastomosis was performed along the longitudinal axis of the gastric. Both anastomosis methods are guaranteed to have an anastomosis length of 5 cm. During the operation, the nasogastric tube (NGT) was routinely placed by anesthesiologist. Peritoneal drainage tubes were separately placed adjacent to the pancreatic-jejunal and biliary-jejunal anastomosis. 

### Postoperative management

All patients were on total parenteral nutrition for at least 3 days. We routinely administered intravenous proton pump inhibitor (PPI) and intravenous pumping of Stilamin (Somatostatin) after surgery. When patient was able to tolerate oral diet, we converted PPI to an oral dosage and Stilamin to subcutaneous injection of Sandostatin. Nasogastric tube was removed within 3 days after surgery according to drainage volume.

### Variables and definition

We created a standardized data form to gather all the relevant information on demographic, preoperative, intraoperative, and postoperative factors. Here are some definitions with clinical diagnostic differences. For preoperative variables, chronic gastropathy was defined as chronic gastritis, gastric ulcer, and reflux esophagitis confirmed by gastroscopy and lasted more than 6 months before hospitalization. Preoperative biliary drainage included percutaneous transhepatic cholangial drainage (PTCD), endoscopic nasobiliary drainage (ENBD), and biliary stent placement by endoscopic retrograde cholangiopancreatography (ERCP). We defined serum total bilirubin < 22.2 μmol/L as normal, 22.2-200 μmol/L as mild jaundice, and ≥ 200 μmol/L as severe jaundice. In this study, all patients with severe jaundice were performed preoperative biliary drainage. A nasogastric tube was inserted preoperatively due to intestinal obstruction caused by tumor overgrowth and compression, namely gastrointestinal decompression. Patients with abnormal liver function had at least one elevation in AST, ALT, GGT, and ALP greater than or equal to two times the upper limit of normal. For intraoperative variables, the definition of minimally invasive pancreaticoduodenectomy was that the completion of tumor resection and digestive tract reconstruction were both performed through laparoscope. Operation time referred to the time from trocar placement or skin incision to complete skin closure. The amount of blood loss, intraoperative plasma and red blood cells transfused were precisely calculated by the anesthesiologist, and then divided into binary variables according to the ROC curve and Youden index, as shown in Table 1. For postoperative complications, patients with intra-abdominal infection must have certain symptoms, abnormal laboratory examination and imaging-confirmed abdominal fluid accumulation. Purulent puncture fluid or positive bacterial tests could also confirm the diagnosis. Incisional infection was defined as: Signs or symptoms of infection, including local redness, swelling, fever, pain, and tenderness; purulent fluid was observed from the incisional tissue; pathogens were cultured from the fluid or tissue of the incision. Moreover, clinically relevant postoperative pancreatic fistula (grade B-C) was defined based on the International Study Group of Pancreatic Surgery (ISGPS) definition [14, 15]. All included intra-abdominal infection and CR-POPF in our article occurred less than POD 14 for grade B and POD 21 for grade C DGE.

**Table 1:** Demographic and perioperative variables in DGE / non-DGE group and Development / Validation set

Variable n (%) or median (IQR)	Whole cohort (*n* = 422)	without CR-DGE (*n* = 328)	with CR-DGE (*n* = 94)	*P*-value	Randomization (3:1 ratio)		*P*-value
					Development set (*n* = 317)	Validation set (*n* = 105)	
Age, years	62.0 (52.0–67.0)	62.0 (52.0–67.0)	60.0 (51.8–66.3)	0.937	61.0 (52.0–67.0)	63 (55–68)	0.299
Sex				0.296			0.195
Male	236 (55.9%)	179 (54.6%)	57 (60.6%)		134 (42.3%)	52 (49.5%)	
Female	186 (44.1%)	149 (45.4%)	37 (39.4%)		183 (57.7%)	53 (50.5%)	
BMI ≥ 22.1 kg/m^2^				0.011			0.783
No	164 (38.9%)	138 (42.1%)	26 (27.7%)		122 (38.5%)	42 (40%)	
Yes	258 (61.1%)	190 (57.9%)	68 (72.3%)		195 (61.5%)	63 (60%)	
Hypertension				0.645			0.263
No	291 (69.0%)	228 (69.5%)	63 (67.0%)		214 (67.5%)	77 (73.3%)	
Yes	131 (31.0%)	100 (30.5%)	31 (33.0%)		103 (32.5%)	28 (26.7%)	
Diabetes mellitus				0.506			0.817
No	325 (77.0%)	255 (77.7%)	70 (74.5%)		245 (77.3%)	80 (76.2%)	
Yes	97 (23.0%)	73 (22.3%)	24 (25.5%)		72 (22.7%)	25 (23.8%)	
Coronary heart disease				0.803			0.061
No	393 (93.1%)	306 (93.3%)	87 (92.6%)		291 (91.8%)	102 (97.1%)	
Yes	29 (6.9%)	22 (6.7%)	7 (7.4%)		26 (8.2%)	3 (2.9%)	
Cerebrovascular disease				0.628			0.741
No	395 (93.6%)	306 (93.3%)	89 (94.7%)		296 (93.4%)	99 (94.3%)	
Yes	27 (6.4%)	22 (6.7%)	5 (5.3%)		21 (6.6%)	6 (5.7%)	
Chronic gastropathy				0.027			0.357
No	380 (90.0%)	301 (91.8%)	79 (84.0%)		283 (89.3%)	97 (92.4%)	
Yes	42 (10.0%)	27 (8.2%)	15 (16.0%)		34 (10.7%)	8 (7.6%)	
Pancreatitis				0.478			0.561
No	397 (94.1%)	310 (94.5%)	87 (92.6%)		297 (93.7%)	100 (95.2%)	
Yes	25 (5.9%)	18 (5.5%)	7 (7.4%)		20 (6.3%)	5 (4.8%)	
Neoadjuvant chemotherapy				0.524			0.533
No	408 (96.7%)	318 (97.0%)	90 (95.7%)		305 (96.2%)	103 (98.1%)	
Yes	14 (3.3%)	10 (3.0%)	4 (4.3%)		12 (3.8%)	2 (1.9%)	
ASA classification				0.519			0.673
1	28 (6.6%)	20 (6.1%)	8 (8.5%)		23 (7.3%)	5 (4.8%)	
2	323 (76.6%)	250 (76.2%)	73 (77.7%)		241 (76.0%)	82 (78.1%)	
3	71 (16.8%)	58 (17.7%)	13 (13.8%)		53 (16.7%)	18 (17.1%)	
Tumor location				0.722			0.069
Pancreatic	277 (65.6%)	219 (66.8%)	58 (61.7%)		197 (62.1%)	80 (76.2%)	
Duodenum	47 (11.1%)	34 (10.4%)	13 (13.8%)		38 (12%)	9 (8.6%)	
Common bile duct	46 (11.0%)	35 (10.7%)	11 (11.7%)		39 (12.3%)	7 (6.7%)	
Vater’s ampulla	52 (12.3%)	40 (12.3%)	12 (12.8%)		43 (13.6%)	9 (8.6%)	
Malignant tumor				0.840			0.815
No	93 (22.0%)	73 (22.3%)	20 (21.3%)		69 (21.8%)	24 (22.9%)	
Yes	329 (78.0%)	255 (77.7%)	74 (78.7%)		248 (78.2%)	81 (77.1%)	
Preoperative biliary drainage				0.125			0.541
No, with normal TB	253 (60.0%)	188 (57.3%)	65 (69.1%)		187 (59.0%)	67 (63.8%)	
No, TB < 200 μmol/L	53 (12.5%)	45 (13.7%)	8 (8.5%)		42 (13.2%)	10 (9.5%)	
Yes, TB ≥ 200 μmol/L	116 (27.5%)	95 (29.0%)	21 (22.4%)		88 (27.8%)	28 (26.7%)	
Preoperative Gastro-intestinal decompression				0.619			0.642
No	416 (98.6%)	324 (98.8%)	92 (97.9%)		313 (98.7%)	103 (98.1%)	
Yes	6 (1.4%)	4 (1.2%)	2 (2.1%)		4 (1.3%)	2 (1.9%)	
Serum albumin ≤ 38, g/L				0.166			0.795
No	379 (89.8%)	291 (88.7%)	88 (93.6%)		284 (89.6%)	95 (90.5%)	
Yes	43 (10.2%)	37 (11.3%)	6 (6.4%)		33 (10.4%)	10 (9.5%)	
Abnormal liver function				0.361			0.399
No	198 (46.9%)	150 (45.7%)	48 (51.1%)		145 (45.7%)	53 (50.5%)	
Yes	224 (53.1%)	178 (54.3%)	46 (48.9%)		172 (54.3%)	52 (49.5%)	
Blood loss ≥ 575 ml				0.201			0.933
No	292 (69.2%)	232 (70.7%)	60 (63.8%)		219 (69.1%)	73 (69.5%)	
Yes	130 (30.8%)	96 (29.3%)	34 (36.2%)		98 (30.9%)	32 (30.5%)	
Plasma transfusion ≥ 400 ml				0.018			0.458
No	285 (67.5%)	231 (70.4%)	54 (57.4%)		211 (66.6%)	74 (70.5%)	
Yes	137 (32.5%)	97 (29.6%)	40 (42.6%)		106 (33.4%)	31 (29.5%)	
RBC transfusion ≥ 1U				0.064			0.940
No	272 (64.5%)	219 (66.8%)	53 (56.4%)		204 (64.4%)	68 (64.8%)	
Yes	150 (35.5%)	109 (33.2%)	41 (43.6%)		113 (35.6%)	37 (35.2%)	
Operation time, hour	6.0 (5.0–7.0)	5.9 (5.0–6.9)	6.1 (5.1–7.2)	0.235	6.0 (5.0–7.0)	6.0 (5.1–7.1)	0.237
Intraoperative fluid input, ml	3900 (3300–4700)	3900 (3300–4700)	4200 (3275–4900)	0.361	3900 (3300–4700)	4000 (3200–4850)	0.992
Pylorus – preserving				0.350			0.540
No	346 (82.0%)	272 (82.9%)	74 (78.7%)		262 (82.6%)	84 (80%)	
Yes	76 (18.0%)	56 (17.1%)	20 (21.3%)		55 (17.4%)	21 (20%)	
Gastrointestinal anastomosis				0.003			0.866
Side-to-side	210 (49.8%)	176 (53.7%)	34 (36.2%)		157 (49.5%)	53 (50.5%)	
End-to-side	212 (50.2%)	152 (46.3%)	60 (63.8%)		160 (50.5%)	52 (49.5%)	
MIPD				0.008			0.757
No	325 (77.0%)	243 (74.1%)	82 (87.2%)		246 (77.6%)	83 (79%)	
Yes	97 (23.0%)	85 (25.9%)	12 (12.8%)		71 (22.4%)	22 (21%)	
Braun’s anastomosis				0.306			0.453
No	389 (92.2%)	300 (91.5%)	89 (94.7%)		294 (92.7%)	95 (90.5%)	
Yes	33 (7.8%)	28 (8.5%)	5 (5.3%)		23 (7.3%)	10 (9.5%)	
Vascular reconstruction				0.720			0.662
No	394 (93.4%)	307 (93.6%)	87 (92.6%)		295 (93.1%)	99 (94.3%)	
Yes	28 (6.6%)	21 (6.4%)	7 (7.4%)		22 (6.9%)	6 (5.7%)	
Jejunostomy				0.763			0.377
No	406 (96.2%)	316 (96.3%)	90 (95.7%)		303 (95.6%)	103 (98.1%)	
Yes	16 (3.8%)	12 (3.7%)	4 (4.3%)		14 (4.4%)	2 (1.9%)	
ICU admission				0.450			0.824
No	201 (47.6%)	153 (46.6%)	48 (51.1%)		150 (47.3%)	51 (48.6%)	
Yes	221 (52.4%)	175 (53.4%)	46 (48.9%)		167 (52.7%)	54 (51.4%)	
Mechanical ventilation, hour	7.5 (5.6–14.9)	7.5 (5.6–15.5)	7.6 (5.9–14.4)	0.913	7.5 (5.5–15.5)	8 (5.8–14.5)	0.607
CR-POPF				0.000			0.335
No	353 (83.6%)	288 (87.8%)	65 (69.1%)		262 (82.6%)	91 (86.7%)	
Yes	69 (16.4%)	40 (12.2%)	29 (30.9%)		55 (17.4%)	14 (13.3%)	
Intra-abdominal infection				0.000			0.460
No	391 (92.7%)	318 (97.0%)	73 (77.7%)		292 (92.1%)	99 (94.3%)	
Yes	31 (7.3%)	10 (3.0%)	21 (22.3%)		25 (7.9%)	6 (5.7%)	
Lung infection				0.050			1.000
No	407 (96.4%)	320 (97.6%)	87 (92.6%)		306 (96.5%)	101 (96.2%)	
Yes	15 (3.6%)	8 (2.4%)	7 (7.4%)		11 (3.5%)	4 (3.8%)	
Urinary infection				1.000			1.000
No	417 (98.8%)	324 (98.8%)	93 (98.9%)		313 (98.7%)	104 (99%)	
Yes	5 (1.2%)	4 (1.2%)	1 (1.1%)		4 (1.3%)	1 (1%)	
Intestinal flora disturbance				0.310			0.602
No	417 (98.8%)	325 (99.1%)	92 (97.9%)		314 (99.1%)	103 (98.1%)	
Yes	5 (1.2%)	3 (0.9%)	2 (2.1%)		3 (0.9%)	2 (1.9%)	
Incisional infection				0.000			1.000
No	411 (97.4%)	326 (99.4%)	85 (90.4%)		309 (97.5%)	102 (97.1%)	
Yes	11 (2.6%)	2 (0.6%)	9 (9.6%)		8 (2.5%)	3 (2.9%)	
CR-DGE	-	-	-	-			0.815
No					248 (78.2%)	81 (77.1%)	
Yes					69 (21.8%)	24 (22.9%)	

*BMI* body mass index, *TB* total bilirubin, *MIPD* minimally invasive pancreaticoduodenectomy, *RBC* red blood cell, *ICU* intensive care unit, *CR-POPF* clinically relevant postoperative pancreatic fistula, *CR-DGE* clinically relevant delayed gastric emptying

### Statistical analysis

Firstly, the 422 enrolled patients were randomly divided (3:1 ratio) into a training cohort with 317 patients and a validation cohort with 105 patients for external validation. Secondly, the LASSO algorithm was applied to filter variables using glmnet R package (version 4.1–4) in the training cohort. We transfer the polytomous variables to binary variables during the LASSO analysis by introducing dummy variables. Ten-fold cross-validation was used to centralize and normalize the included variables with penalty parameter tuning based on minimum criteria and 1 standard error of the minimum criteria (the 1-SE criteria) in the training set. We chose lambda.1se as it gives a model with good performance but the least number of independent variables. Then, a binary multivariable logistic regression analysis was performed to identify significantly contributing variables using the selected factors from LASSO regression analysis. Variables with *p*-value < 0.05 were adopted to develop the nomogram prediction model using rms R package (version 6.3–0). The receiver operation characteristic (ROC) curves and calibration curves were computed using pROC R package (version 1.18.0) and rms R package (version 6.3–0) respectively to evaluate the predictive performance and calibration of the nomogram model. Hosmer–Lemeshow test was used to assess the goodness-of-fit of the model. The clinical usefulness of our nomogram was evaluated by decision curve analysis (DCA) and clinical impact curve (CIC) using rmda R package (version 1.6) by calculating net benefits at different threshold probabilities in two sets. After completing the above procedure, we enrolled another 45 patients as an independent validation cohort to test the clinical diagnosis ability of our nomogram model. In the end, Propensity score matching (PSM) was performed to conduct subgroup analysis with caliper width limited at 0.1.

Categorical variables were exhibited as frequency and percentages. Continuous variables that disobeyed normal distribution were expressed as medians with interquartile ranges (IQR). The Mann–Whitney U test was used for continuous variables, and categorical variables were analyzed using the chi-square test or Fisher’s exact test. A *p*-value of < 0.05 (two-sided) was considered statistically significant. Statistical analysis was performed using SPSS software (version 25.0, IBM Corporation, Armonk, NY, USA) and R software (version 4.1.2; R Foundation for Statistical Computing, Vienna, Austria).

## Results

### Patient characteristics

From January 2019 to December 2021, a total of 507 patients underwent PD and 85 patients were excluded: 31 with intraoperative multiple-organ resection including (21 total pancreatectomy, 4 right hemicolectomy, 9 partial colectomy, 4 partial enterectomy, 1 hepatic segmentectomy, 1 radical distal gastrectomy, 1 left nephrectomy, and adrenalectomy); 7 with major operation history including (1 total gastrectomy, 1 distal gastrectomy, 1 total colectomy, 1 open cholecystectomy and choledochojejunostomy, 1 Beger surgery, 1 sigmoidectomy, 1 hepatectomy for hepatic metastases); 2 with gastrostomy; 1 with post-operative gastric fluid reinfusion; 2 death during hospitalization; 14 with incomplete clinical data and 18 with non-functional delayed gastric emptying. Finally, a total of 422 patients were enrolled in this study. These patients were divided into two groups according to whether clinically relevant DGE (grades B-C) had occurred and randomly placed into the development set or validation set at a ratio of 3:1. Demographic, preoperative, surgical, and postoperative variables were presented in Table 1.

### Predictor selection

**Fig. 1:**
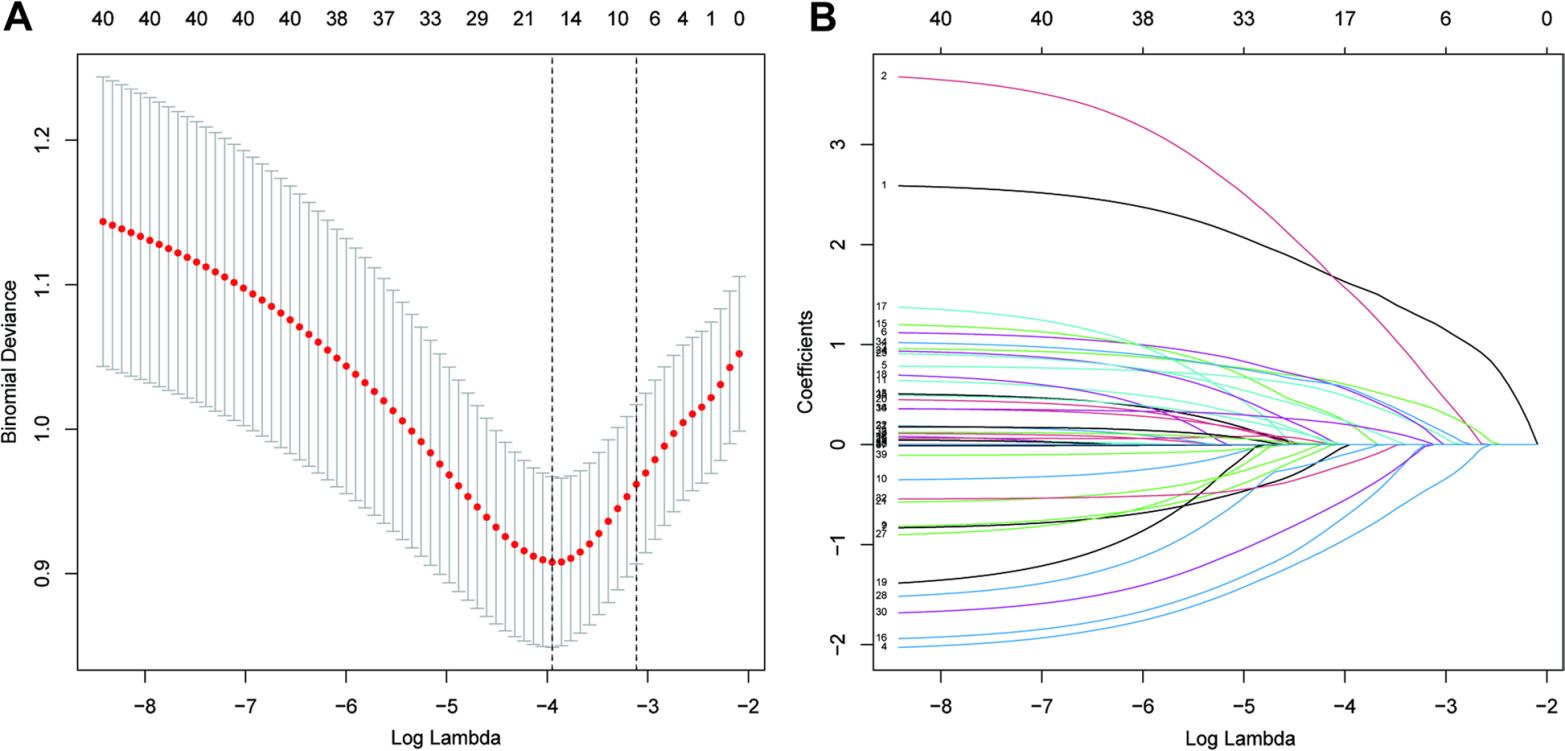
Variables selected by the least absolute shrinkage and selection operator (LASSO) binary logistic regression model with a ten-fold cross-validation

LASSO regression analysis was used to select predictive variables from those shown in Table 1. Seven variables with nonzero coefficients were selected by deriving lambda.1se (Fig. 1), namely intra-abdominal infection, incisional infection, MIPD, gastrointestinal anastomosis, CR-POPF, chronic gastropathy, and intra-operative plasma transfusion volume.

Then, a multivariate logistic regression analysis was performed with these seven selected variables to establish a predictive model (Table 2). All variables were all shown to have statistical significance with *p*-value < 0.05. Hence, none of these variables were removed from the final analysis. For the convenience of calculation, a risk score formula was built to predict the probability of CR-DGE for patients undergoing PD: Logit (P = CR-DGE) = (-2.472) + 2.357 × incisional infection + 2.178 × intra-abdominal infection + (-1.471) × MIPD + 0.836 × End-to-side gastrointestinal anastomosis + 1.043 × Chronic gastropathy + 0.901 × CR-POPF + 0.784 × Plasma transfusion ≥ 400 ml.

**Table 2:** Multivariate logistic regression

	Estimate	SE	Wald	*P*-value	OR	95% confidence interval	
						Lower	Upper
Intro-abdominal infection	2.178	0.541	16.242	0.000	8.831	3.062	25.475
Incisional infection	2.357	1.003	5.520	0.019	10.563	1.478	75.487
CR-POPF	0.901	0.378	5.685	0.017	2.462	1.174	5.164
MIPD	-1.471	0.549	7.176	0.007	0.230	0.078	0.674
End-to-side anastomosis	0.836	0.334	6.277	0.012	2.307	1.200	4.438
History of chronic gastropathy	1.043	0.429	5.910	0.015	2.837	1.224	6.576
Plasma transfusion ≥ 400 ml	0.784	0.316	6.147	0.013	2.191	1.179	4.072

*SE* standard error, *OR* odds ratio, *MIPD* minimally invasive pancreaticoduodenectomy, *CR-POPF* clinically relevant postoperative pancreatic fistula

### Predictive model construction

**Fig. 2:**
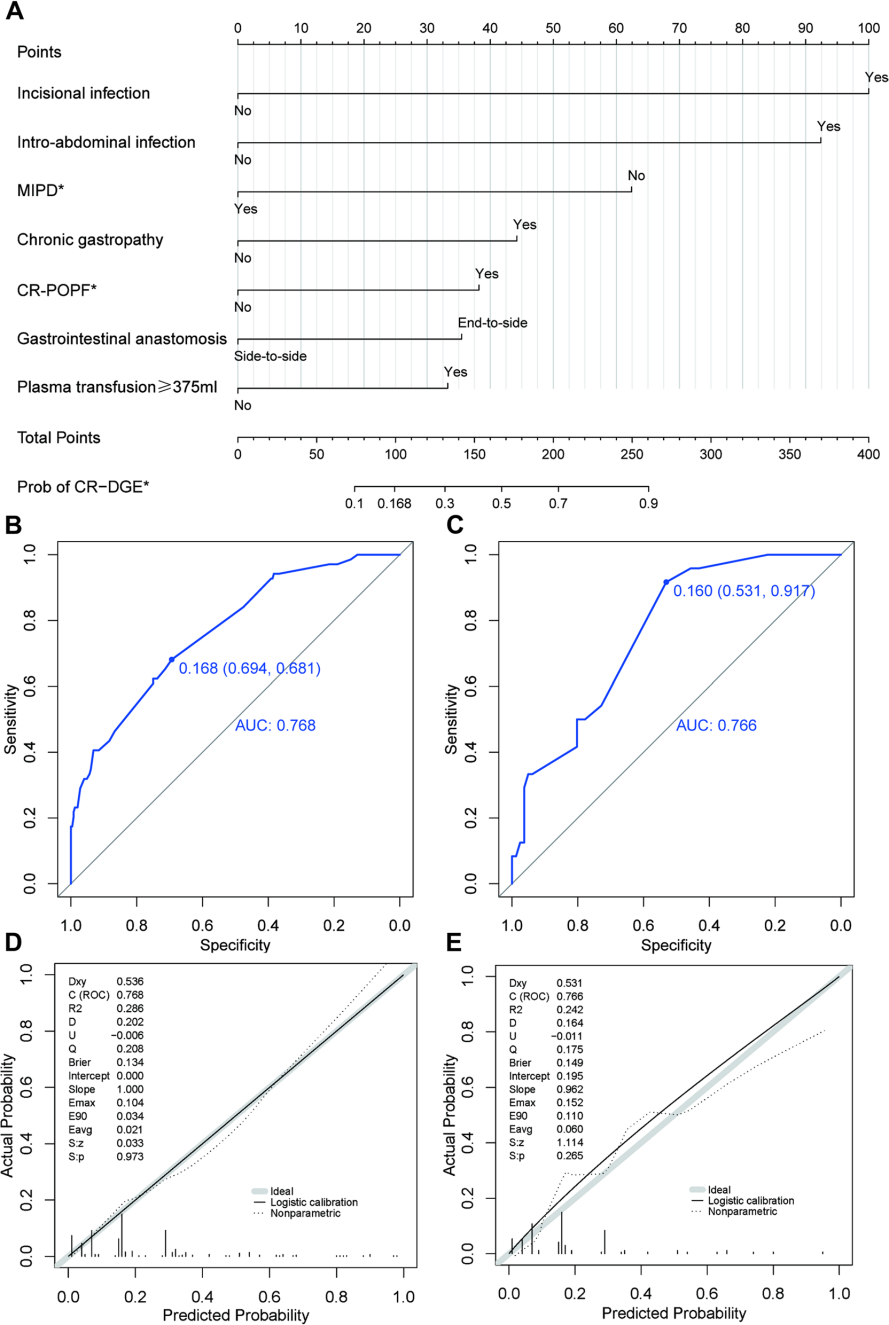
**A** Nomogram for predicting CR-DGE based on the development cohort. MIPD, minimally invasive pancreaticoduodenectomy; CR-POPF, clinically relevant postoperative pancreatic fistula. **B** The receiver operating characteristic curve of the development cohort. **C** The receiver operating characteristic curve of the validation cohort. **D** The calibration curve of the development cohort. **E** The calibration curve of the validation cohort

The predictive model was visualized as a nomogram (Fig. 2A), and the risk probability of CR-DGE would be predicted quantitatively using this model. For each patient, users need to draw virtual vertical lines from each variable to the “Points” axis, identify the points for each variable, and sum these scores to calculate the total point along with the probability of CR-DGE. The ROC analysis showed that areas under the curve (AUC) of the development and validation cohorts were 0.768 (95% CI, 0.706–0.830) and 0.766 (95% CI, 0.671–0.861), respectively (Fig. 2B and C). The Youden-derived cutoff value for the nomogram was 0.168, corresponding to a total point value of 99. At this point, the nomogram had a sensitivity of 68.1%, specificity of 69.4%, NPV of 88.7%, PPV of 38.2%, and accuracy of 69.1%. The calibration curve and Hosmer–Lemeshow test were used to calibrate the predictive model. The well-fitted calibration curves in both the development and validation sets were shown in Fig. 2D and E, demonstrating great prediction accuracy between the actual probability and the predicted probability using the nomogram model. Detailed parameters were placed in the calibration curve plots. As demonstrated in the Hosmer–Lemeshow test, the predicted and actual probabilities were highly consistent (Development set, *P* = 0.957; Validation set, *P* = 0.521).

**Fig. 3:**
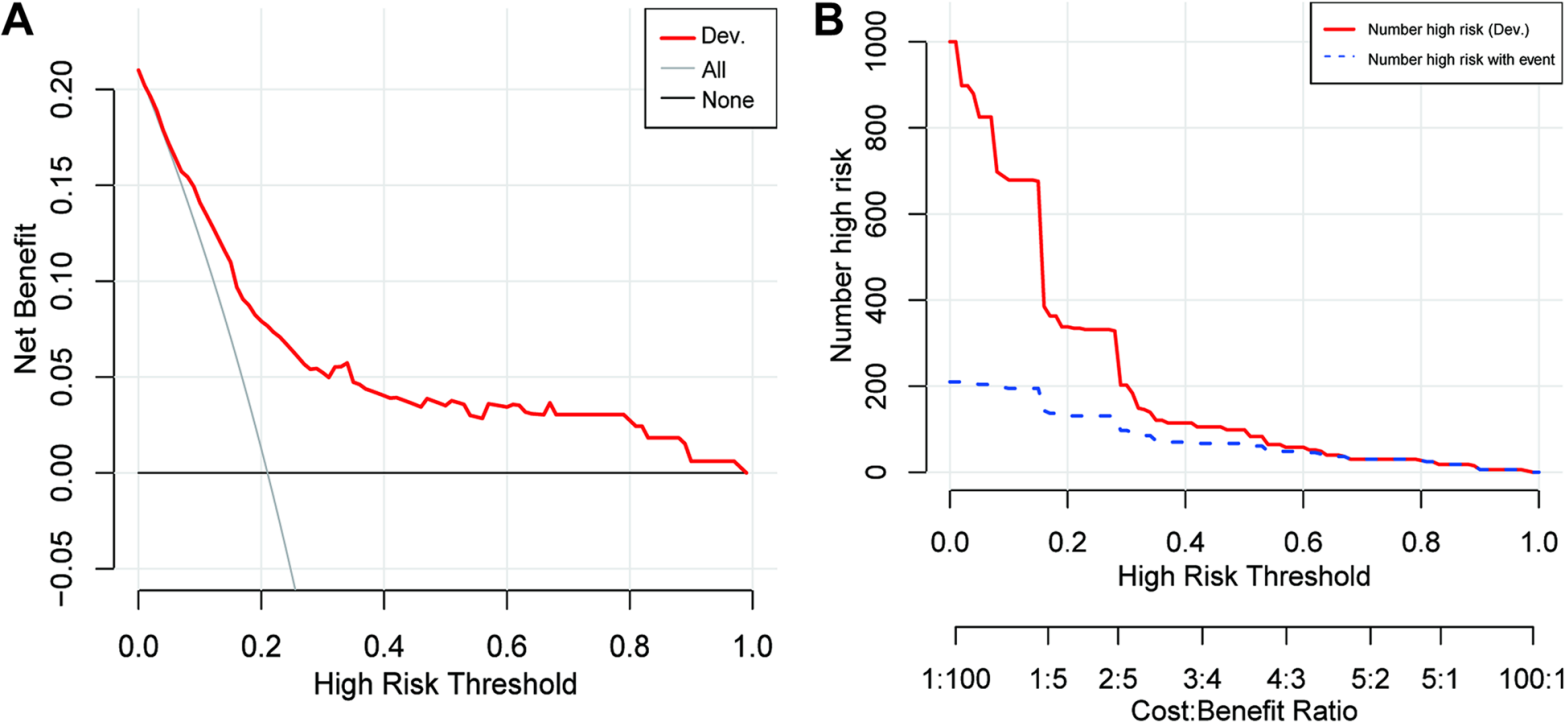
**A** Decision curve analysis of development cohort; ‘All’ refers to that all patients have CR-DGE and ‘none’ to that no patient has CR-DGE. **B** clinical impact curve of development cohort (population size = 1000). The red solid line refers to the total patients who would be regarded as high risk for each risk threshold. The blue dashed line refers to that how many of those would be real CR-DGE patients. Dev, development set

Furthermore, we performed decision curve analysis (DCA) and clinical impact curves (CIC) in the development cohort (Fig. 3), which all showed that our nomogram model had great net clinical benefit and clinical utility.

**Fig. 4:**
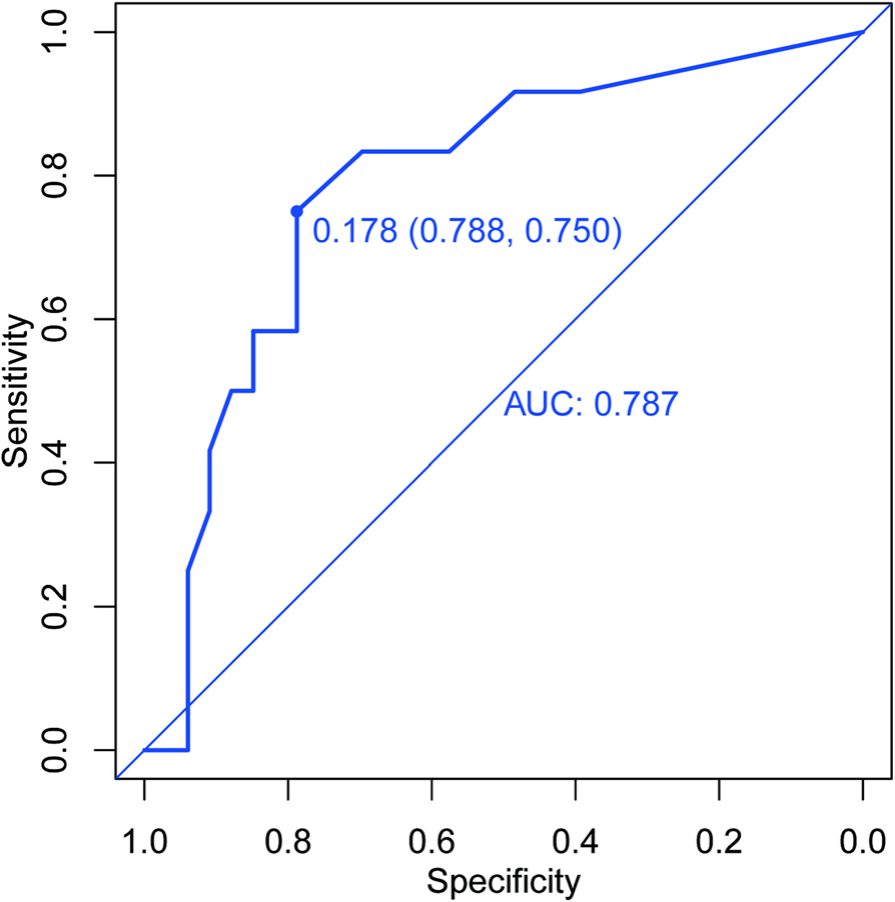
ROC curve of the independent validation cohort

Lastly, we collected another 45 consecutive patients following the above exclusion criteria from January 2022 to March 2022 in the same hospital as an independent validation cohort. ROC curve was plotted in Fig. 4. Areas under the curve (AUC) was 0.787 (95% CI, 0.633–0.940). This further confirmed the practicability and universality of our model.

### Subgroup analysis

Specifically, the type of gastrointestinal anastomosis varied depending on surgical options, termed as gastrojejunostomy (GJ) in PrPD and duodenojejunostomy (DJ) in PPPD. Hence, we divided the patients underwent PrPD and PPPD into GJ and DJ group, respectively. Propensity score matching (PSM) was used to avoid confounding differences between the GJ and DJ group by balancing all variables except CR-DGE. As shown in Table 3, after PSM, patients who underwent side-to-side gastrojejunostomy in PrPD cohort (*n* = 346) had significantly lower incidence of CR-DGE compare with end-to-side gastrojejunostomy (15.8% vs 28.4%; *P* = 0.036). Similarly, in PPPD cohort (*n* = 76), only 5 patients underwent side-to-side duodenojejunostomy and none had CR-DGE (0.0%). In contrast, 20 of the other 71 patients who underwent end-to-side duodenojejunostomy patients suffered from CR-DGE (28.1%). Unfortunately, there was no statistical difference between these two in PPPD cohort because of the limited sample size. Hence, large sample studies are needed to confirm the superiority of side-to-side duodenojejunostomy in the prevention of DGE.

**Table 3:** Variables of the whole and matched cohorts in patients with PrPD

Variable n (%) or median (IQR)	PrPD cohort (*n* = 346)		*P*-value	Matched cohort in PrPD cohort (*n* = 190)		*P*-value
	Side-to-side group (*n* = 141)	End-to-side group (*n* = 205)		Side-to-side group (*n* = 95)	End-to-side group (*n* = 95)	
Age, years	63.0 (56.0–68.0)	63.0 (53.5–68.0)	0.440	63.0 (52.0–68.0)	63.0 (50.0–68.0)	0.997
Sex			0.587			0.663
Male	118 (42.4%)	77 (54.6%)		49 (51.6%)	52 (54.7%)	
Female	87 (57.6%)	64 (45.4%)		46 (48.4%)	43 (45.3%)	
BMI ≥ 22.1 kg/m^2^			0.587			1.000
No	83 (40.5%)	53 (37.6%)		38 (40.0%)	38 (40.0%)	
Yes	122 (59.5%)	88 (62.4%)		57 (60.0%)	57 (60.0%)	
Hypertension			0.043			0.545
No	145 (70.7%)	85 (60.3%)		59 (62.1%)	63 (66.3%)	
Yes	60 (29.3%)	56 (39.7%)		36 (37.9%)	32 (33.7%)	
Diabetes mellitus			0.238			0.855
No	161 (78.5%)	103 (73.0%)		77 (81.1%)	76 (80.0%)	
Yes	44 (21.5%)	38 (27.0%)		18 (18.9%)	19 (20.0%)	
Coronary heart disease			0.560			0.579
No	191 (93.2%)	129 (91.5%)		87 (91.6%)	89 (93.7%)	
Yes	14 (6.8%)	12 (8.5%)		8 (8.4%)	6 (6.3%)	
Cerebrovascular disease			0.616			0.351
No	189 (92.2%)	132 (93.6%)		91 (95.8%)	88 (92.6%)	
Yes	16 (7.8%)	9 (6.4%)		4 (4.2%)	7 (7.4%)	
Chronic gastropathy			0.924			0.468
No	184 (89.8%)	127 (90.1%)		84 (88.4%)	87 (91.6%)	
Yes	21 (10.2%)	14 (9.9%)		11 (11.6%)	8 (8.4%)	
Pancreatitis			0.071			0.774
No	197 (96.1%)	129 (91.5%)		88 (92.6%)	89 (93.7%)	
Yes	8 (3.9%)	12 (8.5%)		7 (7.4%)	6 (6.3%)	
Neoadjuvant chemotherapy			0.344			0.678
No	195 (95.1%)	137 (97.2%)		90 (94.7%)	91 (95.8%)	
Yes	10 (4.9%)	4 (2.8%)		5 (5.3%)	4 (4.2%)	
ASA classification			0.428			1.000
1	6 (2.9%)	8 (5.7%)		4 (4.2%)	6 (6.3%)	
2	161 (78.5%)	106 (75.2%)		76 (80.0%)	71 (74.7%)	
3	38 (18.5%)	27 (19.1%)		15 (15.8%)	18 (18.9%)	
Tumor location			0.499			0.967
Pancreatic	127 (62.0%)	96 (68.1%)		64 (67.4%)	61 (64.2%)	
Duodenum	25 (12.2%)	14 (9.9%)		10 (10%)	12 (12.6%)	
Common bile duct	23 (11.2%)	17 (12.1%)		11 (11.6%)	12 (12.6%)	
Vater’s ampulla	30 (14.6%)	14 (9.9%)		10 (10.5%)	10 (10.5%)	
Malignant tumor			0.439			1.000
No	30 (14.6%)	25 (17.7%)		19 (20.0%)	19 (20.0%)	
Yes	175 (85.4%)	116 (82.3%)		76 (80.0%)	76 (80.0%)	
Preoperative biliary drainage			0.564			0.791
No, with normal TB	111 (54.1%)	81 (57.4%)		52 (54.7%)	52 (54.7%)	
No, TB < 200 μmol/L	28 (13.7%)	25 (15.6%)		16 (16.8%)	13 (13.7%)	
Yes, TB ≥ 200 μmol/L	66 (32.2%)	50 (27.0%)		27 (28.4%)	30 (31.6%)	
Preoperative Gastro-intestinal decompression			0.691			1.000
No	202 (98.5%)	138 (97.9%)		93 (97.9%)	93 (97.9%)	
Yes	3 (1.5%)	3 (2.1%)		2 (2.1%)	2 (2.1%)	
Serum albumin ≤ 38 g/L			0.282			1.000
No	185 (90.2%)	122 (86.5%)		83 (87.4%)	83 (87.4%)	
Yes	20 (9.8%)	19 (13.5%)		12 (12.6%)	12 (12.6%)	
Abnormal liver function			0.900			0.381
No	93 (45.4%)	63 (44.7%)		45 (47.4%)	39 (41.1%)	
Yes	112 (54.6%)	78 (55.3%)		50 (52.6%)	56 (58.9%)	
Blood loss ≥ 575 ml			0.369			0.871
No	133 (64.9%)	98 (69.5%)		69 (72.6%)	68 (71.6%)	
Yes	72 (35.1%)	43 (30.5%)		26 (27.4%)	27 (28.4%)	
Plasma transfusion ≥ 400 ml			0.001			0.539
No	145 (70.7%)	76 (53.9%)		65 (68.4%)	61 (64.2%)	
Yes	60 (29.3%)	65 (46.1%)		30 (31.6%)	34 (35.8%)	
RBC transfusion ≥ 1U			0.478			0.453
No	127 (62.0%)	82 (58.2%)		62 (65.3%)	57 (60.0%)	
Yes	78 (38.0%)	59 (41.8%)		33 (34.7%)	38 (40.0%)	
Operation time, hour	6.0 (5.0–6.8)	5.9 (5.0–7.0)	0.543	6.0 (5.2–6.6)	5.0 (5.9–7.0)	0.266
Intraoperative fluid input, ml	3900 (3300–4700)	3800 (3200–4650)	0.041	3900 (3300–4700)	3800 (3200–4500)	0.330
Gastrointestinal anastomosis			0.003			0.866
Side-to-side	176 (53.7%)	34 (36.2%)		157 (49.5%)	53 (50.5%)	
End-to-side	152 (46.3%)	60 (63.8%)		160 (50.5%)	52 (49.5%)	
MIPD			0.000			0.364
No	140 (68.3%)	131 (92.9%)		82 (86.3%)	86 (90.5%)	
Yes	65 (31.7%)	10 (7.1%)		13 (13.7%)	9 (9.5%)	
Braun’s anastomosis			0.389			0.601
No	190 (92.7%)	127 (90.1%)		86 (90.5%)	88 (92.6%)	
Yes	15 (7.3%)	14 (9.9%)		9 (9.5%)	7 (7.4%)	
Vascular reconstruction			0.235			0.579
No	193 (94.1%)	128 (90.8%)		89 (93.7%)	87 (91.6%)	
Yes	12 (5.9%)	13 (9.2%)		6 (6.3%)	8 (8.4%)	
Jejunostomy			0.035			1.000
No	203 (99.0%)	134 (95.0%)		93 (97.9%)	94 (98.9%)	
Yes	2 (1.0%)	7 (5.0%)		2 (2.1%)	1 (1.1%)	
ICU admission			0.033			0.884
No	91 (44.4%)	79 (56.0%)		53 (55.8%)	52 (54.7%)	
Yes	114 (55.6%)	62 (44.0%)		42 (44.2%)	43 (45.3%)	
Mechanical ventilation, hour	8.0 (5.8–15.3)	7.0 (5.5–14.3)	0.159	7.0 (6.0–12.0)	7.0 (5.5–13.8)	0.826
CR-POPF			0.287			1.000
No	167 (81.5%)	121 (85.8%)		83 (87.4%)	83 (87.4%)	
Yes	38 (18.5%)	20 (14.2%)		12 (12.6%)	12 (12.6%)	
Intra-abdominal infection			0.056			0.721
No	185 (90.2%)	135 (95.7%)		92 (96.8%)	90 (94.7%)	
Yes	20 (9.8%)	6 (4.3%)		3 (3.2%)	5 (5.3%)	
Lung infection			0.133			1.000
No	194 (94.6%)	138 (97.9%)		94 (98.9%)	94 (98.9%)	
Yes	11 (5.4%)	3 (2.1%)		1 (1.1%)	1 (1.1%)	
Urinary infection			1.000			1.000
No	202 (98.5%)	139 (98.6%)		94 (98.9%)	93 (97.9%)	
Yes	3 (1.5%)	2 (1.4%)		1 (1.1%)	2 (2.1%)	
Intestinal flora disturbance			0.082			1.000
No	200 (97.6%)	141 (100.0%)		95 (100.0%)	95 (100.0%)	
Yes	5 (2.4%)	0 (0.0%)		0 (0.0%)	0 (0.0%)	
Incisional infection			0.211			1.000
No	196 (95.6%)	139 (98.6%)		94 (98.9%)	93 (97.9%)	
Yes	9 (4.4%)	2 (1.4%)		1 (1.1%)	2 (2.1%)	
CR-DGE			0.009			**0.036**
No	171 (83.4%)	101 (71.6%)		80 (84.2%)	68 (71.6%)	
Yes	34 (16.6%)	40 (28.4%)		15 (15.8%)	27 (28.4%)	

*PrPD* pylorus-resecting pancreatoduodenectomy, *BMI* body mass index, *TB* total bilirubin, *MIPD* minimally invasive pancreaticoduodenectomy, *RBC* red blood cell, *ICU* intensive care unit, *CR-POPF* clinically relevant postoperative pancreatic fistula, *CR-DGE* clinically relevant delayed gastric emptying

### Development of a simplified scale

**Fig. 5:**
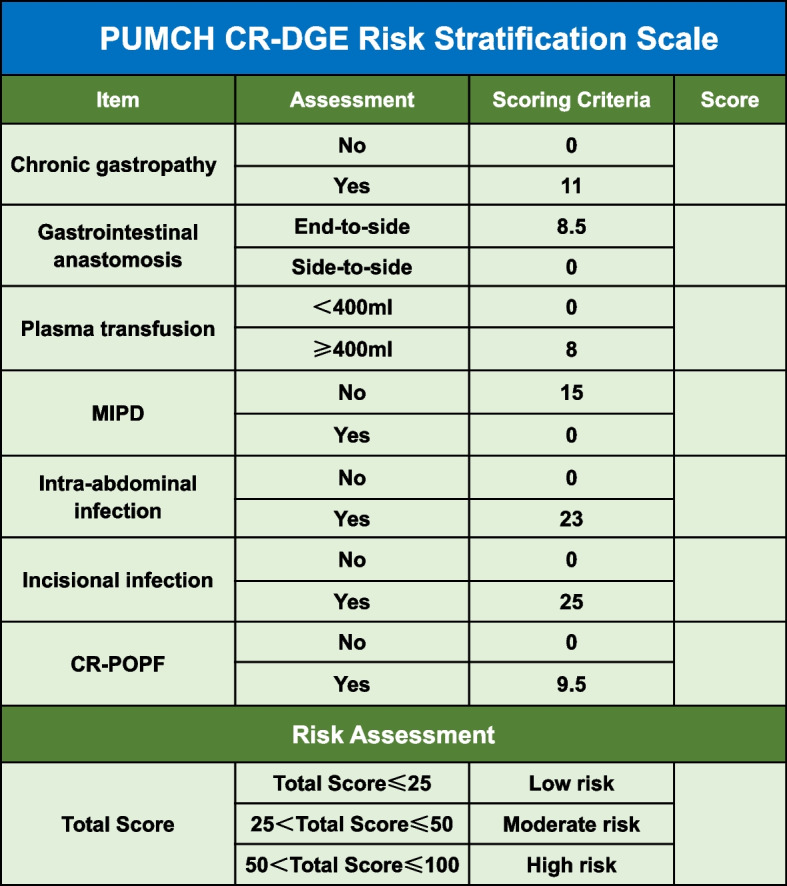
CR-DGE Risk Stratification Scale of Peking Union Medical College Hospital (PUMCH). MIPD, minimally invasive pancreaticoduodenectomy; CR-POPF, clinically relevant postoperative pancreatic fistula; PUMCH, Peking Union Medical College Hospital

To make our predictive model briefer and more practical in the clinical application, we simplified the scoring system of the nomogram and established a new scale, named CR-DGE Risk Stratification Scale of Peking Union Medical College Hospital (PUMCH) (Fig. 5). The lowest and highest scores on this scale are 0 and 100, respectively. We defined an overall score of less than the cutoff value of the nomogram as low risk. If the total score is ≤ 25, surgeons can regard the PD patient as CR-DGE low risk. If the total score of a patient is > 25, he/she has a relatively higher risk of suffering CR-DGE.

## Discussion

Delayed gastric emptying remains one of the most common complications after pancreaticoduodenectomy. The incidence of CR-DGE (grade B-C) in this study was 22.2% according to the ISGPS definitions. Risk stratification for patients after PD decreased the length of stay and improved early postoperative outcomes [16, 17]. As reported by Arango [18], the implementation of risk-stratified pancreatectomy clinical pathways (RSPCPs), which classified patients as high risk or low risk based on their risk of developing POPF, could reduce the incidence of DGE from 41 to 21% in high-risk group and 36% to 15% in low-risk group. There have been many newly published studies related to the predictors of DGE as shown in Table 4. However, no studies have yielded a risk stratification for PD patients based on postoperative DGE. Thus, we attempted to develop a nomogram to stratify the risk level of DGE.

**Table 4:** Recently reported studies in predicting risk factors and protective factors for DGE

Author, year	Country	Type	Research period	Sample size	Outcome variable	Risk factors	Protective factors
Herrera, 2019 [19]	Spain	RCT	2013–2016	64	Grade A-C	Male gender, Intro-abdominal complications, Preoperative malnutrition, Hemorrhage	NA
Watanabe, 2020 [20]	Japan	Retrospective	2009–2018	281	Grade B-C	POPF, TACC > 110 mm	NA
Cai, 2020 [2]	China	Retrospective	2009–2018	308	Grade B-C	PPPD, Biliary leakage, Intra-abdominal infection, Diabetes	NA
Lee, 2020 [21]	South Korea	Retrospective	2010–2016	115	Grade A-C	PPPD, POPF grade B-C	NA
Snyder, 2020 [22]	USA	ACS-NSQIP database	2014–2016	10,249	Grade B-C	Age > 65 years, Male sex, BMI > 30, ASA ≥ 3, PPPD, Longer operative time (> 356 min)	Preoperative chemotherapy
Quero, 2021 [23]	Italy	Prospective	2019–2020	121	Grade B-C	ASA ≥ 3, Horizontal gastrojejunostomy orientation, Flow angle > 30° (Efferent jejunal limb and the stomach), Clavien-Dindo complications ≥ 3	NA
Varghese, 2021 [24]	New Zealand	Meta-Analysis on 24 RCTs	1999- 2020	2526	Grade B-C	PPPD	Antecolic gastrojejunostomy, Braun’s anastomosis
Arango, 2021 [18]	USA	Prospective	2011–2019	724	Grade A-C	PPPD, Intra-abdominal abscess, Non-white patient	Implementation of RSPCPs
Fahlbusch, 2022 [25]	Germany	StuDoQ|Pancreas database	2014–2018	5,080	Grade A-C	Higher age, Longer operative time, Pancreaticogastrostomy, POPF	NA
Werba 2022 [1]	USA	ACS-NSQIP database	2014–2018	15,154	Grade B-C	Age > 70 years, COPD, Smoker, ASA ≥ 3, Male sex and Preoperative RBC transfusion, PPPD, Pancreaticogastrostomy, Adhesiolysis, Jejunostomy, Vascular reconstruction	Biliary stent placement, Active smoking

*POPF* postoperative pancreatic fistula, *TACC* thickness of the abdominal cavity at the level of the celiac axis, *PPPD* pylorus—preserving pancreaticoduodenectomy, *BMI* body mass index, *RSPCPs* risk-stratified pancreatectomy clinical pathways, *COPD* chronic obstructive pulmonary disease, *RBC* red blood cell, *RCT* randomized controlled trial

Several independent risk factors for DGE were identified in our nomogram, including previous history of chronic gastropathy (OR = 2.837, 95% CI: 1.224–6.576, *P* = 0.015), intraoperative plasma transfusion volume (OR = 2.191, 95% CI: 1.179–4.072, *P* = 0.013), end-to-side gastrojejunostomy (OR = 2.307, 95% CI: 1.200–4.438, *P* = 0.012), and postoperative complications such as intra-abdominal infection (OR = 8.831, 95% CI: 3.062–25.475, *P *= 0.000), incisional infection (OR = 0.019, 95% CI: 1.478–75.487, *P* = 0.019), and CR-POPF (OR = 2.462, 95% CI: 1.174–5.164, *P *= 0.017). Additionally, minimally invasive operation (OR = 0.230, 95% CI: 0.078–0.674, *P* = 0.007) was considered to be a protective factor for DGE. The pathogenesis of DGE after PD is not fully understood. Current hypotheses include: Partial removal of the digestive tract reduces the secretion of gastrointestinal hormones such as gastrin and motilin, promoting gastric emptying; motor incoordination due to digestive tract reconstruction and vagotomy [10]; surgical injury of gastric tissue along with extended peri-gastric devascularization reduces blood supply; reflux of digestive juices into the stomach leads to inflammatory edema and hormonal disorders [2]; remnant stomach cannot generate effective basic electrical rhythm and contraction waves due to the lack of interstitial cells of Cajal (ICC) [10, 26, 27]; lack of migrating motor complexes (MMC) originating from the antrum and duodenum [28]; psychological factors [29].

Several recently published meta-analyses [29–32] have demonstrated the feasibility and safety of MIPD, including less intraoperative blood loss, more thorough lymph node dissection, and a shorter duration of hospitalization. As for postoperative DGE, some [31] supported that MIPD could reduce its incidence while others [29, 33] did not. Further multicenter RCTs are required to confirm and update the results of these analyses. Several articles [34–36] have also demonstrated the superiority of side-to-side gastrojejunostomy for PD patients in preventing DGE, which matched our conclusion. The possible mechanism is as follows: Firstly, the anastomosis was made on the posterior gastric wall along the greater curvature. Therefore, gastric contents can be easily and quickly drained into the jejunum. Secondly, the application of endovascular gastrointestinal anastomosis stapler in side-to-side method could more uniformly secure the anastomotic lumen opening and prevent anastomotic stenosis. Additionally, anastomotic patency was no longer restricted by lumen diameter compared to end-to-side method, leading to a better-controlled length of anastomosis. Doctors also could adjust the anastomotic position according to blood supply and length of remanent small intestine.

Our nomogram listed incisional infection and intro-abdominal infection as the two highest risk factors affecting DGE. The role of intro-abdominal infection in promoting DGE has been verified in previous articles [8, 18, 19, 37]. However, the effect of incisional infection on the development of DGE still lacks literature support. We assume that it might be associated with prolonged application of antibiotics, immobilization, and inflammatory response. As reported by Mirrielees et al. [38], the most common complications of pancreaticoduodenectomy were pancreatic fistula and delayed gastric emptying. In a multicenter, prospective analysis of 711 patients receiving pancreaticoduodenectomy or total pancreatectomy, Parmar et al. [39] found that pancreatic fistula was highly related to the incidence of DGE. Similarly, Li and colleagues [21] reported that clinically relevant postoperative pancreatic fistula (grade B-C) was an independent risk factor that predicted DGE. In contrast, neither of the present studies support that DGE can cause pancreatic fistula.

Interestingly, excessive intraoperative plasma transfusion was found to be an independent risk factor of CR-DGE in our nomogram. Transfusions of allogenic blood could elevate blood arginase and nitric oxide synthase, resulting in a transient immunosuppressive state and activating infection-related processes [40]. Although there are no other studies demonstrating the correlation between blood transfusion and DGE, the following studies all showed its negative impact postoperatively. Marincola et al. [41] reported that blood transfusion was related to increasing rates of postoperative complications including wound disruption, intra-abdominal abscess, deep surgical site infection (SSI), and urinary infection for patients who underwent pancreatic neuroendocrine tumors (PNETs) resection. Besides, blood transfusion could also delay anastomosis healing, leading to high risk of anastomotic leakage [42]. In another retrospective study of 6869 patients underwent pancreaticoduodenectomy, perioperative blood transfusion was independently associated with postoperative infection, including pneumonia, sepsis, and septic shock.

Some retrospective single-center analyses and meta-analyses [24, 43, 44] found that Braun’s anastomosis, which enabled bile and pancreatic fluid to bypass anastomosis from afferent jejunal loop to efferent loop, had a significant impact on preventing mechanical afferent loop obstruction and reducing the incidence of DGE. However, a randomized controlled trial led by Fujieda et al. [45] produced the opposite result. Our study did not fully reveal its clinical practicability toward DGE because all patients with postoperative mechanical intestinal obstruction were excluded. Consistently, patients’ age, gender, BMI, diabetes mellitus, ASA classification, and operative time have been reported to be associated with the occurrence of DGE [1, 11, 22]. Contrary to these reports, our study did not find evidence to support them. We also found no significant difference between the PPPD and PrPD groups in the incidence of CR-DGE.

Overall, the development of CR-DGE after PD is linked to a variety of factors. Apart from a history of chronic gastropathy, none of the preoperative factors previously reported such as albumin level, BMI, age, ASA score, and biliary drainage were associated with CR-DGE. Therefore, this article mainly focused on the predictive value of intraoperative and postoperative factors for CR-DGE. Side-to-side anastomosis, minimally invasive surgery, less blood transfusion, early prevention and treatment for pancreatic fistula, and infection-related complications might reduce the incidence of CR-DGE.

Although our research included many preoperative indicators, the major limitation of this study was that some factors listed in the nomogram could only be obtained post-operatively, such as CR-POPF, intra-abdominal infection, and incisional infection, resulting in a lack of preoperative predictive efficacy. Future research should therefore aim to develop preoperative predictive models. Also, the nomogram was developed and validated in a single center for Chinese populations and lacked external validation. Lastly, this study was designed in a retrospective database with a relatively small sample size and therefore selection bias may have been present.

## Conclusion

The present study successfully established a nomogram of clinically relevant delayed gastric emptying after pancreaticoduodenectomy and created a simplified scale for risk stratification and early intervention.

## Data Availability

All data analyzed during this study are available from the corresponding author on reasonable request.

## Abbreviations

DGE: Delayed gastric emptying
PD: Pancreaticoduodenectomy
LASSO: Least absolute shrinkage and selection operator
CR-DGE: Clinically relevant delayed gastric emptying
ROC: Receiver operating characteristic curve
DCA: Decision curve analysis
CIC: Clinical impact curve
MIPD: Minimally invasive pancreaticoduodenectomy
CR-POPF: Clinically relevant postoperative pancreatic fistula
ERAS: Enhanced recovery after surgery
NGT: Nasogastric tube
PSM: Propensity score matching
